# Supplementary material for: Nuclear Organization during Hepatogenesis in Zebrafish Requires Uhrf1
Source: Genes (Basel). 2021 Jul 16;12(7):1081. doi: 10.3390/genes12071081 (PMC8304062; doi:10.3390/genes12071081)
Supplement: Supplementary file 1 [file genes-12-01081-s001.zip › SUPPLEMENTARY FILES.pdf]

Supplementary Materials and Methods:

Trypan blue exclusion method for cell viability

Trypan blue exclusion test was used to measure the viability of zebrafish 5 dpf *Tg(fabp10a: CAAX-EGFP)* liver cells from for ATAC-Seq library generation. Live cells possess intact cell membranes, and exclude Trypan blue while dead cells do not. So, viable cells have a clear cytoplasm whereas a nonviable cells have a dark blue cytoplasm. We used the TC20 automated cell counter (Bio-Rad Inc, USA) for assessing the viability and number of live cells. A total volume of liver cells in PBS was 200 μl for each clutch. For this procedure, a 1:1 dilution of liver cells in PBS suspension were made in 0.4% trypan blue stain (Biorad, #1450015) in an Eppendorf tube (10 μl of cells added to 10 μl of trypan blue). 10 microliters of the mixture were then loaded into the opening of the TC20 counting slide (Biorad, #1450015). The slide was then inserted into the TC20 instrument. The cell counter automatically detects the presence of the counting slide and starts to count the number of cells. The result is displayed as number of cells/ml and the corresponding viability. From this, the number of viable cells in the total volume of 200μl was calculated. A minimum of 95% viability was considered to proceed with ATAC-Seq library preparation. Cells were counted within 5 minutes of mixing trypan blue.

Supplementary figures:

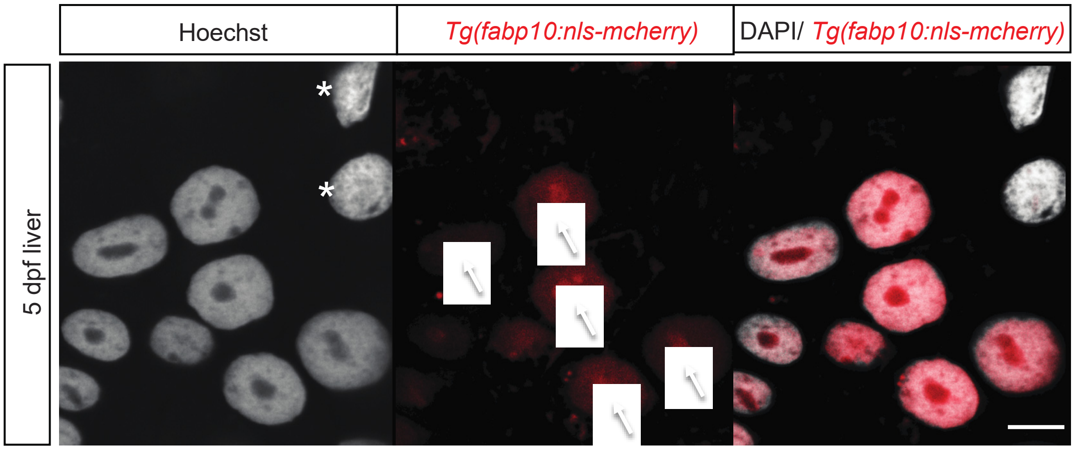

Figure S1: The hepatocyte nuclei marked with nuclear localized fluorescent protein. Representative confocal image of Hoechst stained Tg(*fabp10a:nls-mcherry)* hepatocytes from 120 hpf larvae. White arrows mark nls-cherry enriched nucleolus in hepatocytes. Asterix (*) mark non-hepatocytes. Scale bar: 6μm

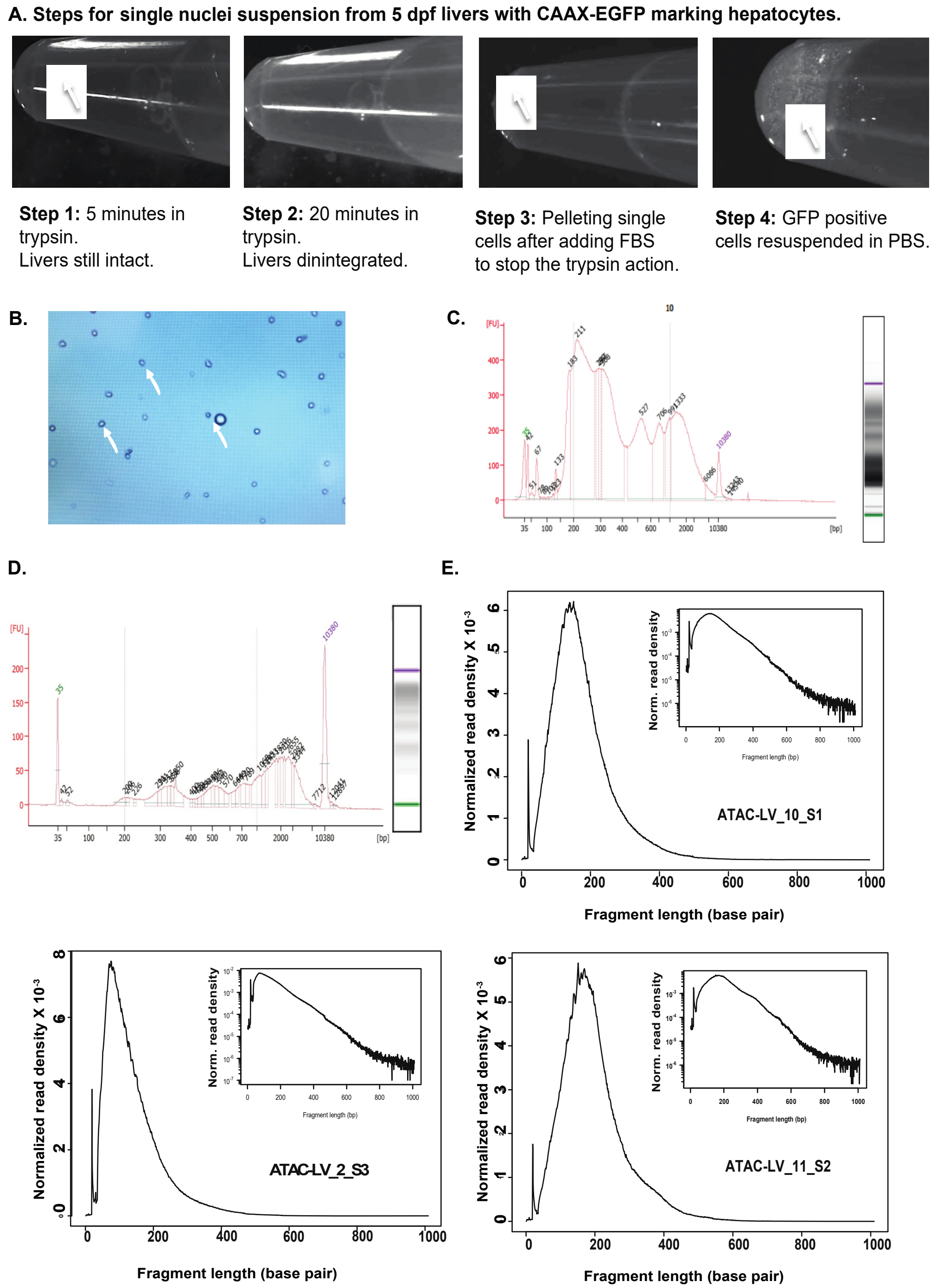

Figure S2: Generation of ATAC-Seq libraries from 120 hpf zebrafish livers
A. Steps involved in single cell isolation of cells from 120 hpf livers**. B.** Viability of cells checked by trypan blue exclusion staining (clear cytoplasm represents live cells and blue cytoplasm for dead cells) **C., D.** Bioanalyzer profile of 120 hpf ATAC-Seq library from 10,000 live cells and 500 cells respectively. **E.** Fragment length distribution after alignment in 3 biological replicates are all around 200 bp close to the length of mono-nuleosome 180 bp.

A.

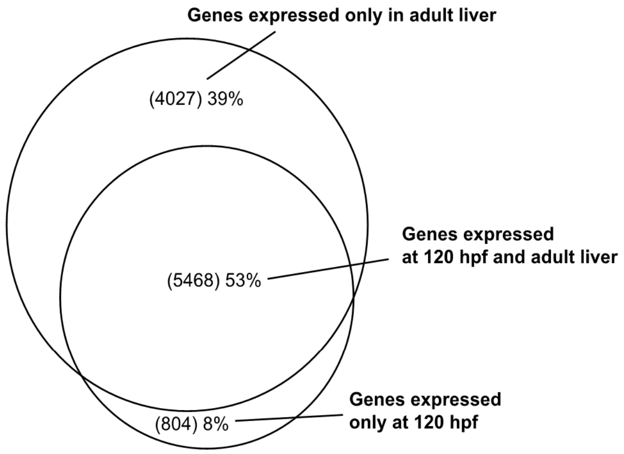

B.

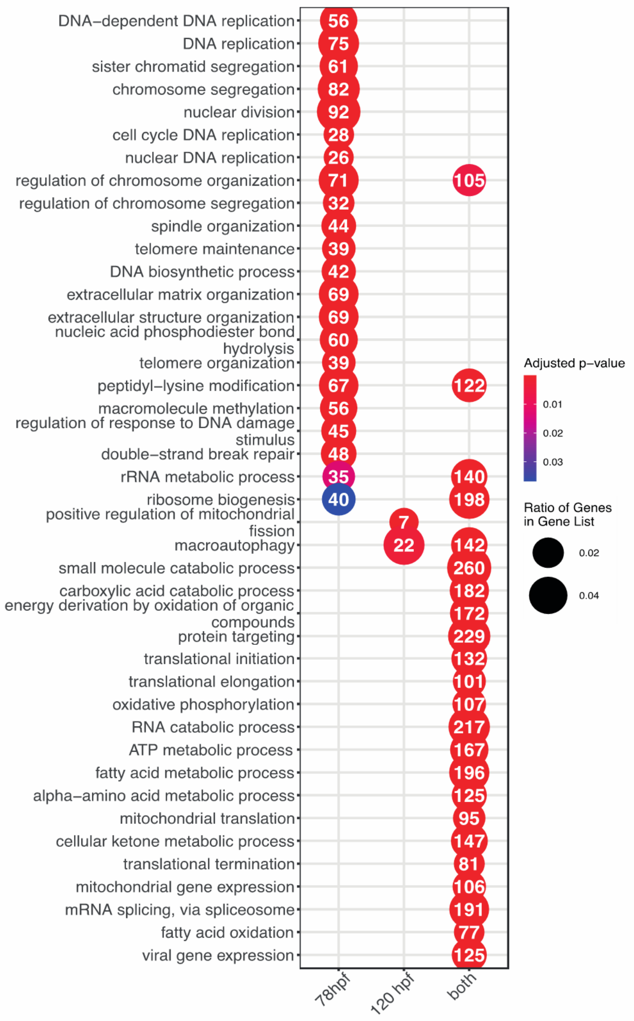

Figure S3: Gene expression comparison between 78 and 120 hpf livers
A. Venn diagram of genes expressed in adult liver (TPM >5) compared to genes expressed at 120 hpf (FPKM >5). Numbers in brackets represent the number of genes in each group and the percentages of genes in each group are shown. **B.** Gene Ontology of Biological Processes of gene categories showed in Figure 4A. For each group the 20 most enriched terms are plotted.

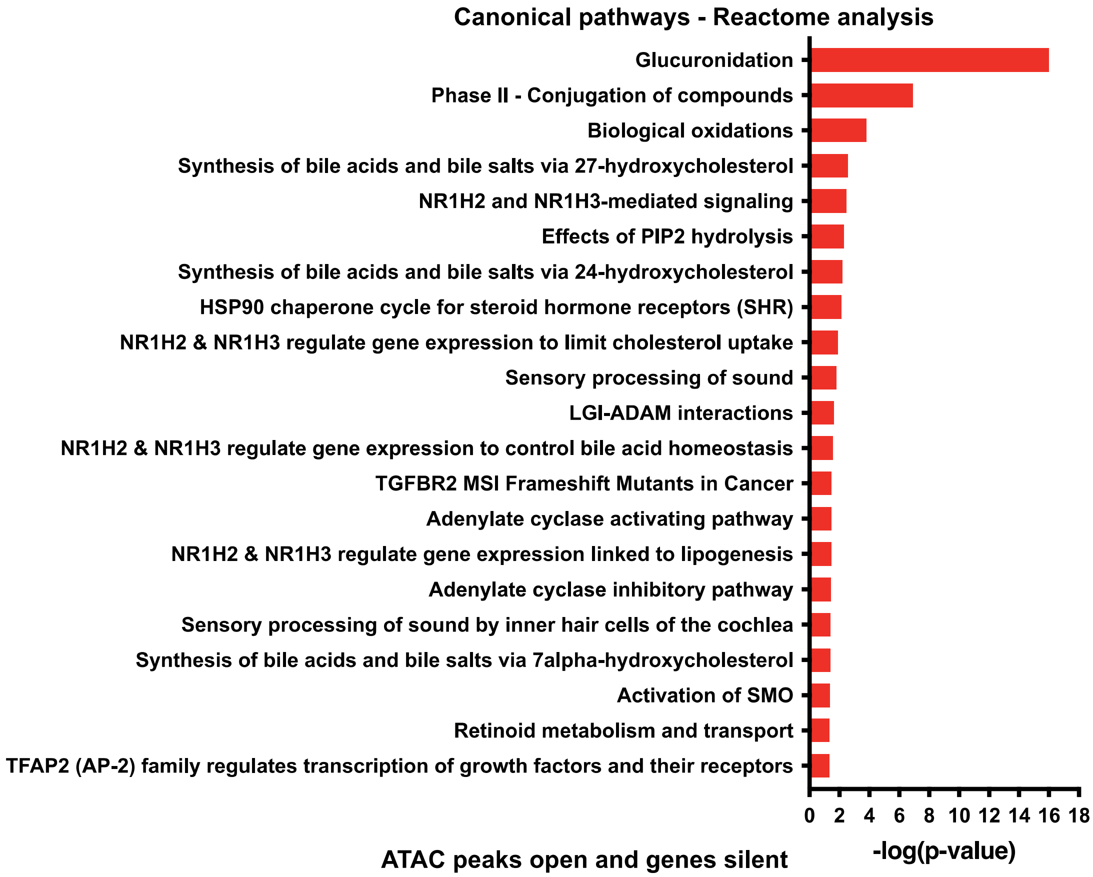

Figure S4: Genes residing in closed chromatin which remain silent at 120 hpf are mainly involved in biliary function

Gene Ontology of Biological Processes of ATAC-Seq peaks not expressed at 120 hpf. The 20 most enriched terms are plotted.

A

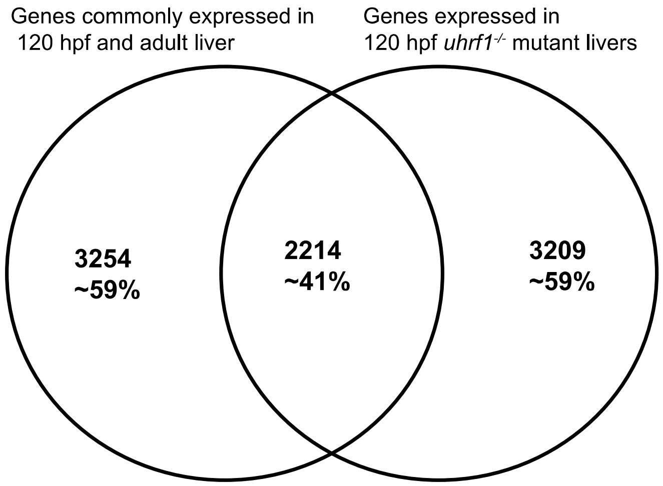

B

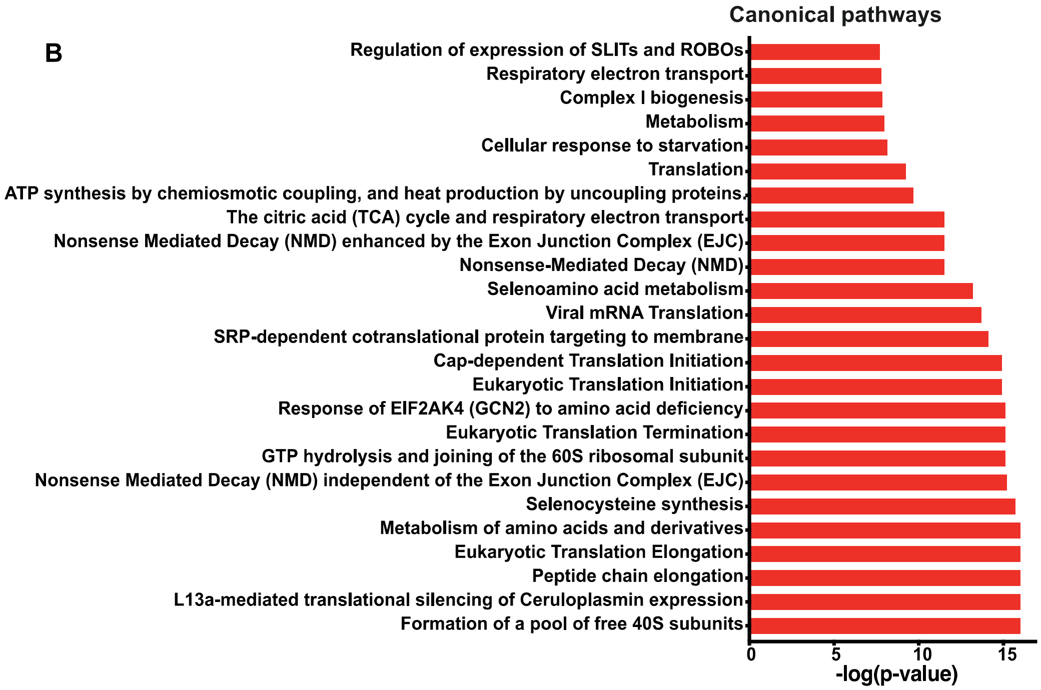

Figure S5: Liver differentiation is not affected in 120 hpf *uhrf1$^{-/-}$* livers
Venn diagram comparing the overlap of gene expression between control livers of 2 stages (120 hpf control livers, adult livers) and 120 hpf *uhrf1$^{-/-}$* mutant livers. B. Gene Ontology of the common genes between the 2 datasets (2214 genes). For each group the 25 most enriched terms were chosen and plotted

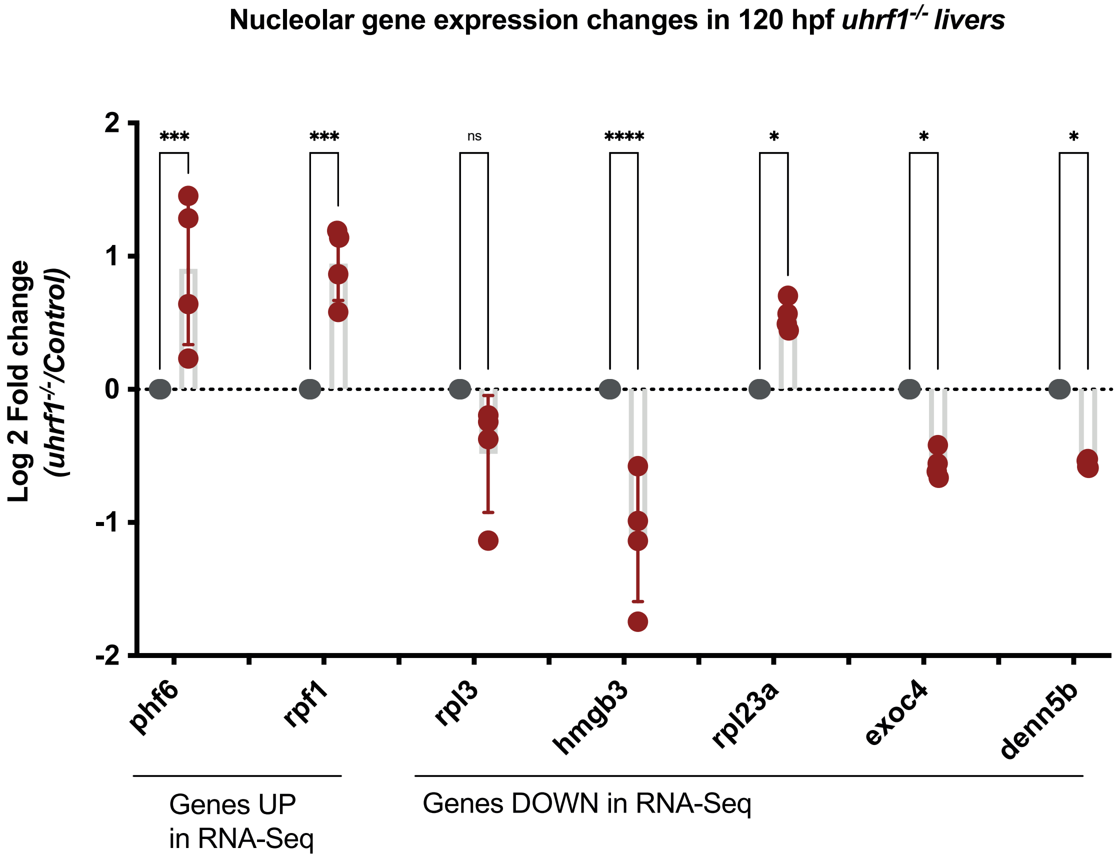

Figure S6

Figure S6: Re-confirmation of nucleolar gene expression changes by Q-PCR in 120 hpf *uhrf1*$^{-/-}$ livers

qPCR analysis of nucleolar genes in 120 hpf *uhrf1*$^{-/-}$ livers compared to their controls. Rplp0 is used as loading controls and the delta-delta Ct (DDCt) values were calculated by normalization to rplp0 and control sibling for each individual clutch. Lines in the graph represents the median. Statistical significance is calculated by paired t-test. **$p < 0.05$, ***$p < 0.005$, ****$p < 0.001$.

Table S1: List of primers used for real-time QPCR analysis in Figure 6, Figure S6

Table S2: Sequencing data set summary (see attached Excel spreadsheet “Table S2.xlsx”)

Table S3: DESeq summary of the RNA-Seq comparison of 78 hpf to 120 hpf control livers
